# Outcomes of Older Adults Admitted to the ICU Following Trauma: A Scoping Review Identifying the Nonagenarian Evidence Gap

**DOI:** 10.7759/cureus.102425

**Published:** 2026-01-27

**Authors:** Harry W Botta, Nattaya Raykateeraroj, Jemin Suh, Dong-Kyu Lee, Laurence Weinberg

**Affiliations:** 1 Department of Anaesthesiology, Austin Health, Melbourne, AUS; 2 Department of Anesthesiology, Faculty of Medicine, Siriraj Hospital, Mahidol University, Bangkok, THA; 3 Department of Anesthesiology, Austin Health, Melbourne, AUS; 4 Department of Anesthesiology and Pain Medicine, Dongguk University Ilsan Hospital, Goyang, PRK; 5 Department of Critical Care, University of Melbourne, Melbourne, AUS

**Keywords:** elderly population, elderly trauma, icu mortality rate, icu resources, oldest old

## Abstract

As populations age, trauma-related intensive care unit (ICU) admissions among older adults are increasing, including nonagenarians who are increasingly offered ICU-level care. However, ICU outcomes for nonagenarians remain essentially undescribed, and existing trauma literature largely aggregates patients aged 65 years and older without age-specific analyses of the oldest cohorts. This scoping review sought to characterize ICU outcomes after traumatic injury in elderly and oldest adults, and to determine whether any evidence specifically addresses nonagenarians.

We conducted a scoping review following the Arksey-O’Malley framework and the PRISMA-ScR guidance. MEDLINE (Ovid), the Cochrane Library, and gray literature sources were searched from inception to April 10, 2025. Studies were eligible if they reported mortality outcomes for adults aged ≥65 years admitted to the ICU following trauma. Data on age thresholds, injury severity scores (ISS), intubation, ICU length of stay, complications, surgical procedures, and trauma mechanisms were extracted by one reviewer and independently verified by a second. Given substantial clinical and methodological heterogeneity, findings were summarized using descriptive statistics only; a sample-size-weighted average mortality was calculated as a descriptive summary measure.

Of 226 records screened, 7 retrospective cohort studies met the inclusion criteria. No study analyzed nonagenarians (≥90 years) as a distinct subgroup; included cohorts were defined using thresholds of >65, >70, >75, or >80 years. In-hospital mortality ranged from 3.9% to 20.2% across heterogeneous elderly ICU trauma populations, with higher mortality consistently observed in patients with higher ISS and those experiencing unplanned ICU admission or ICU readmission. A sample-size-weighted average in-hospital mortality of 10.5% was observed across all studies; this value is descriptive and must be interpreted cautiously in view of variations in age structure, injury patterns, admission criteria, and eras of care. Falls were the predominant trauma mechanism (44.8%-67.3%), followed by motor vehicle collisions. Median ISS ranged from 9 to 19, and median ICU length of stay from 2 to 10 days, with longer stays associated with mechanical ventilation and complications such as respiratory failure, pneumonia, and sepsis. Intubation rates ranged from 5% to 61%, and surgical intervention rates from 0.8% to 30.7%, most commonly chest drainage and orthopedic procedures.

Current evidence on ICU outcomes after trauma in elderly patients is derived from heterogeneous cohorts aged ≥65-80+ years and does not provide nonagenarian-specific data. Within these broader elderly ICU trauma populations, higher ISS and unplanned ICU admission are consistently associated with increased in-hospital mortality, and respiratory complications are common. However, the absence of dedicated analyses for patients aged ≥90 years, the lack of long-term functional and quality-of-life outcomes, and the retrospective design of all available studies substantially limit inferences for nonagenarians. Future research should prioritize prospective, age-stratified cohorts including nonagenarians, incorporate frailty and baseline function as key prognostic variables, and report standardized mortality and functional outcomes to inform ICU triage, prognostication, and resource allocation for the oldest trauma patients.

## Introduction and background

The age distribution of populations worldwide is increasingly shifting towards older age groups, with a growing proportion of individuals surviving into their eighth, ninth, and tenth decades of life [1]. In Australia, the proportion of the population aged 65 years and over rose from 12.4% to 16.3% between 2000 and 2020 [2], and in the United States, the number of older adults is projected to increase from approximately 49 million in 2016 to an estimated 73 million by 2030 [3]. This demographic transformation is accompanied by a rising volume of geriatric trauma presentations, including patients requiring intensive care unit (ICU) admission after injury [4]. For trauma and critical care services, this trend poses substantial challenges, as older patients frequently present with complex care needs driven by multimorbidity, polypharmacy, frailty, and diminished physiological reserve [5]. Trauma remains a major contributor to mortality in this group and is a leading cause of death among older adults in the United States [6].

To date, much of the geriatric trauma literature has focused on blunt thoracic trauma and rib fractures, where older adults experience disproportionately higher mortality than younger individuals with comparable injuries [7]. These findings have underpinned the development and adoption of ICU admission protocols for older adults with blunt thoracic trauma, particularly those with multiple rib fractures, at both institutional and statewide levels [8]. By contrast, evidence is far more limited for elderly trauma patients requiring ICU-level care for other injury patterns or general trauma, despite octogenarians and nonagenarians representing a rapidly expanding subset of the older adult population at high risk of adverse outcomes after major trauma [9]. Although some studies have compared outcomes in older versus younger trauma cohorts [10,11], very few have examined the oldest adults specifically, and almost none have undertaken age-stratified analyses that isolate nonagenarians as a distinct group.

Importantly, no prior review has synthesized ICU outcomes following traumatic injury in older adults in general, nor has any review specifically addressed the oldest cohorts, such as octogenarians or nonagenarians. The study by Oshima et al. [12] compared outcomes for trauma patients aged over 80 years with those of younger patients admitted to the ICU but did not extend analyses to nonagenarians (≥90 years) or evaluate long-term functional outcomes. In parallel, the continuing expansion of the oldest age groups and the growing constraints on ICU capacity and healthcare resources make it increasingly important to understand not only survival, but also the longer-term consequences and value of ICU admission for very old trauma patients [13]. Without robust data, clinicians and health systems lack an evidence base to support prognostication, shared decision-making, and the development of age- and injury-specific ICU triage and management protocols.

In this context, a scoping review is methodologically appropriate to map the breadth, nature, and gaps in the existing evidence rather than to estimate pooled effect sizes. This scoping review, therefore, aimed to synthesize the current literature on ICU outcomes for elderly trauma patients, with particular attention to the oldest adults and to determining whether any data specifically address nonagenarians. It was guided by the following questions: (1) To what extent and in what nature of published literature examines ICU outcomes following trauma in patients aged ≥90 years? (2) What outcomes, such as mortality, injury severity, complications, length of stay, and interventions, have been reported for older adults (≥65 years) admitted to the ICU after trauma? (3) What key knowledge gaps exist regarding nonagenarian trauma ICU outcomes? (4) How might the available data inform ICU admission criteria, resource allocation, and future research priorities for the oldest trauma patients? By evaluating injury patterns, ICU and hospital length of stay, complications, and mortality across elderly ICU trauma cohorts, this review seeks to provide a structured overview of the current evidence landscape and to identify critical gaps that must be addressed to improve critical care decision-making for this vulnerable and rapidly growing population.

## Review

Methodology

Funding, Protocol Registration, and Reporting

**Figure 1:**
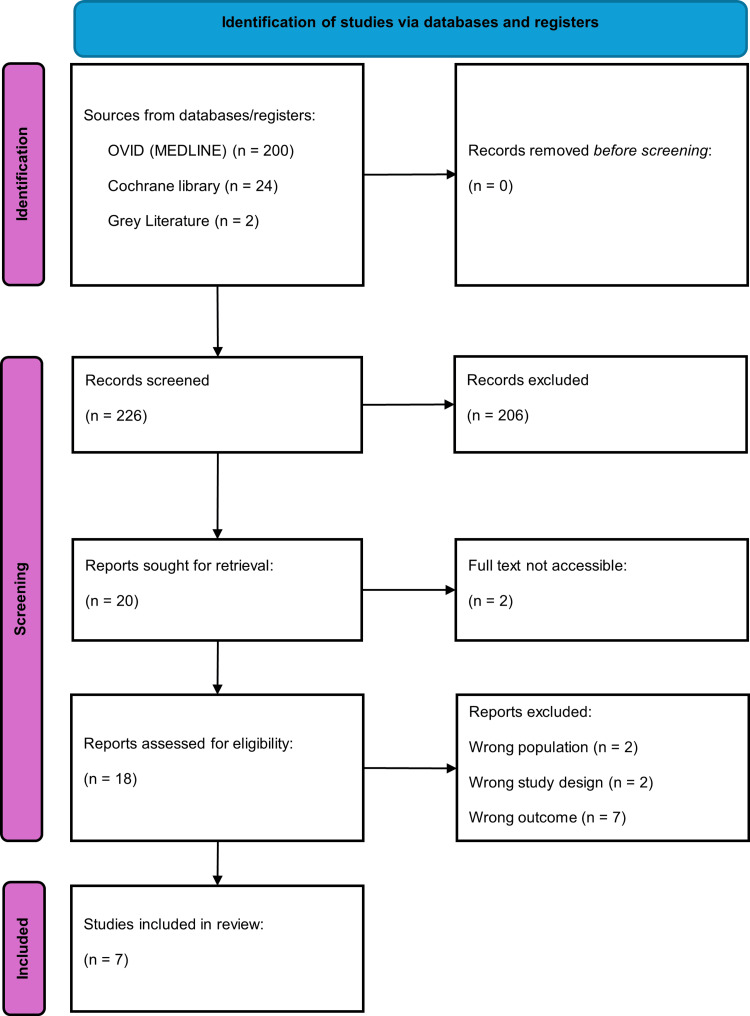
Study flow diagram.

No external funding was obtained for this scoping review. A review protocol was not prospectively registered or published before conducting the review, which we acknowledge as a limitation that may introduce risk of selection or reporting bias. Our institution strongly encourages protocol registration for all evidence synthesis projects; however, PROSPERO (International Prospective Register of Systematic Reviews) does not accept scoping review registrations. To mitigate the risk of bias, we adhered strictly to the Arksey and O’Malley framework [14], as refined by Levac et al. [15], and the Preferred Reporting Items for Systematic Reviews and Meta-Analyses Extension for Scoping Reviews (PRISMA-ScR) guidelines [16]. Inclusion and exclusion criteria were defined a priori using a population, concept, and context (PCC) framework before commencing screening. Two reviewers independently screened all titles, abstracts, and full texts. We maintained comprehensive documentation of search strategies (Appendix) and screening decisions (Figure 1) and reported all included studies with extracted data in structured tables, without selective reporting.

Information Sources and Search Strategy

MEDLINE (accessed via OVID) and the Cochrane Library were searched from database inception to April 10, 2025. MEDLINE was selected as the primary database because it comprehensively indexes core clinical, trauma, and critical care literature, including major trauma and critical care journals relevant to this review. The Cochrane Library was included to identify relevant systematic reviews, controlled trials, and evidence syntheses. We acknowledge that additional databases such as Embase, Scopus, Web of Science, and CINAHL would have enhanced search comprehensiveness; however, feasibility constraints at the time of the review precluded their inclusion. PubMed was not searched separately as it substantially overlaps with MEDLINE content, and our OVID MEDLINE search captured indexed literature comprehensively as of April 10, 2025.

To mitigate the risk of missing relevant studies, we employed several supplementary strategies: (1) systematic grey literature searching via Google and Google Scholar; (2) manual screening of reference lists from all included articles; and (3) manual screening of reference lists from relevant reviews and clinical guidelines identified during the search process. Grey literature was searched systematically on February 15, 2025, using structured search term combinations, including: "nonagenarian trauma ICU outcomes," "oldest adults trauma intensive care," "elderly trauma ICU admission over 90," "geriatric trauma ICU mortality," and Boolean combinations of ["nonagenarian" OR "oldest old" OR "very elderly" OR "≥90 years"] AND ["trauma" OR "injury"] AND ["ICU" OR "intensive care"] AND ["outcomes" OR "mortality"]. For each search, the first 10 pages of results (approximately 100 results per search) were screened by title for relevance using the same inclusion and exclusion criteria applied to database searches. Potentially relevant items underwent full-text review when accessible. Results were documented in screening records. Two potentially eligible records were identified through grey literature searches; one was excluded for not reporting mortality outcomes, and the other was a conference abstract subsequently published as a full article already captured in the MEDLINE search. We acknowledge that grey literature searching using general search engines has inherent reproducibility limitations due to personalized algorithms, geographical variations in results, and temporal changes in indexed content.

Keywords relating to older adults, oldest adults, ICU, intensive care, trauma, and injury were mapped to relevant Medical Subject Headings (MeSH) and combined with free-text terms to ensure broad search coverage. The complete search strategy as executed in MEDLINE (OVID) is presented in the Appendix. An initial search was completed on February 10, 2025, with a final updated search conducted on April 10, 2025, to capture any additional literature published in the interim period.

Eligibility Criteria: Publication Types

All published randomized controlled trials, prospective or retrospective cohort studies, cross-sectional studies, case series, and grey literature reporting on older or oldest adults admitted to ICU following traumatic injury were eligible for inclusion. Gray literature was included due to the anticipated low volume of studies examining the target population. Protocols for planned studies, conference abstracts without accompanying full publications, editorials, narrative reviews, dissertations, and studies for which full text was not available in English were excluded. Case reports (single patients) were excluded due to limited generalizability.

Eligibility Criteria: Study Population

Anticipating a paucity of evidence specifically examining nonagenarians (≥90 years), we adopted a broad age inclusion criterion: studies were eligible if they reported outcomes for adults aged ≥65 years, ≥70 years, ≥75 years, or ≥80 years admitted to ICU following traumatic injury. We explicitly acknowledge that this broad age threshold introduces substantial heterogeneity, as included studies might examine populations with median ages ranging from the late 60s to the mid-80s. This heterogeneity was deemed necessary for several reasons: (1) preliminary scoping indicated that no studies specifically examined nonagenarian ICU trauma outcomes as a distinct cohort, and restricting inclusion to nonagenarian-only studies would have resulted in an empty review; (2) studies with broad age ranges (e.g., ≥65 years) theoretically include nonagenarians within their cohorts even if subgroup analyses were not performed, thereby allowing us to identify the evidence base within which nonagenarians are embedded but not separately examined; (3) by including studies across the spectrum of elderly age definitions, we could demonstrate the progressive narrowing of available evidence as age thresholds increase, contextualizing the absence of nonagenarian-specific data within the broader pattern of age-stratified research; and (4) this approach aligns with scoping review objectives of mapping the extent and nature of available evidence and identifying gaps, rather than estimating precise treatment effects for clinical application. We recognize in the limitations that findings from cohorts with median ages in the 70s cannot be assumed to generalize to nonagenarians.

Studies examining any type of traumatic injury, including isolated or combined head, thoracic, abdominal, limb, or pelvic trauma, were eligible, if patients were admitted to ICU following injury. This broad injury inclusion criterion was intentional to determine whether nonagenarian-specific evidence exists for any trauma type and to identify whether certain injury patterns have received disproportionate research attention. Publications that did not relate to ICU admission in the context of traumatic injury were excluded.

Eligibility Criteria: Outcomes

The primary outcome of interest was mortality associated with ICU admission following traumatic injury. Studies that did not report mortality were excluded. From included studies, additional data on intubation rates, surgical interventions, ICU and hospital length of stay, and complications during admission were extracted where reported.

Screening Process

Two independent reviewers (HB and JS) screened all titles and abstracts, followed by full-text assessment of potentially eligible articles, using predefined inclusion and exclusion criteria. Discrepancies were resolved through discussion, with a third reviewer (LW) consulted when consensus could not be reached. Screening followed a three-stage approach. In stage one, titles and abstracts were assessed and categorized as "include," "exclude," or "maybe." In stage two, full texts of records categorized as "maybe" were retrieved and reviewed to determine final eligibility, with non-compliant studies reclassified as "exclude." In stage three, full-text screening with systematic application of eligibility criteria was applied to all articles classified as "include." EndNote (Clarivate, Philadelphia, PA) was used to manage records and retrieve full-text articles. Studies for which full text could not be obtained were excluded. The screening process and results are summarized in a PRISMA flow diagram (Figure 1).

Data Extraction

Using the final set of studies meeting eligibility criteria, a standardized data extraction spreadsheet was developed a priori. Extracted data included: study information (authors, year of publication, years of data collection, study design, country, setting); sample size and patient demographics (age threshold, median or mean age, sex distribution); clinical characteristics (injury severity score (ISS), comorbidities, frailty measures if reported); trauma details (mechanism of injury, anatomic injury type); ICU admission pathway (planned vs. unplanned); outcomes (in-hospital mortality, ICU and hospital length of stay, intubation, mechanical ventilation, complications, surgical procedures, discharge destination); and risk of bias assessment. Data extraction was performed by one author (HB) using the pre-specified template, with independent verification of all extracted items by a second author (LW) against the source publications. Discrepancies or uncertainties were discussed among the full author group until consensus was reached. We acknowledge that single-reviewer extraction with verification introduces greater risk of extraction error compared with fully independent dual extraction, although this risk is partially mitigated by the descriptive nature of the synthesis and the use of a structured extraction form.

Risk of Bias Assessment

Risk of bias was assessed independently by two reviewers (HB, JS) using the Risk Of Bias In Non-randomized Studies of Interventions tool (ROBINS-I, version 2, 2024) [17] and the Scottish Intercollegiate Guidelines Network (SIGN) checklist for cohort studies [18]. Disagreements were resolved by discussion or by consultation with a third reviewer (NR). Quality assessments informed interpretation of individual study findings; however, consistent with scoping review methodology, we did not use quality scores to weight studies or exclude them from synthesis.

Data Synthesis

Data were displayed in tabular and graphical formats and synthesized using descriptive and narrative statistics. Given the substantial clinical and methodological heterogeneity across the included studies, encompassing diverse age thresholds (≥65 to ≥80 years), injury types (isolated rib fractures versus general polytrauma), care settings (single-center versus multicenter, United States, Japan, Spain), admission protocols (protocolized ICU admission for specific injuries versus unplanned or reactive ICU admission), injury severity (median ISS ranging from 9 to 19), and temporal contexts (data collection spanning 1987 to 2019), we considered formal meta-analysis inappropriate and potentially misleading. Scoping reviews are explicitly designed to map the extent and nature of available evidence rather than to synthesize pooled effect estimates, as articulated in the Arksey and O'Malley framework [14] and PRISMA-ScR guidance [16].

Quantitative synthesis is characteristic of systematic reviews addressing focused questions with homogeneous populations and interventions; the current evidence landscape for elderly trauma ICU outcomes does not meet these conditions. Accordingly, quantitative data analysis was restricted to descriptive statistics conducted using Microsoft Excel (Microsoft Corporation, Redmond, WA). Summary statistics are reported as counts, proportions, medians with interquartile ranges (IQRs), or means with standard deviations (SDs), as originally reported in source studies. A sample-size-weighted average in-hospital mortality was calculated as a descriptive summary measure using the formula: weighted average = Σ(mortality rate × sample size)/Σ(sample size). This approach weights larger studies more heavily and provides readers with a single summary statistic describing central tendency across studies; however, we emphasize that this is a descriptive statistic, not a meta-analytic pooled estimate, and must be interpreted cautiously considering the marked heterogeneity. Study characteristics and patient demographics were organized into structured summary tables to facilitate comparison of outcomes across studies.

Results

Study Selection

The literature search identified 226 records for screening. Of these, 20 were assessed as potentially eligible ("include" or "maybe") based on title and abstract review. Full texts could not be retrieved for two records. Of the remaining 18 articles undergoing full-text assessment, 11 were excluded: two examined populations outside the inclusion criteria, two did not meet study design requirements, and seven did not report the required mortality outcome. Seven retrospective cohort studies met all eligibility criteria and were included in the review. The screening and study selection process is depicted in Figure 1.

Study Characteristics and Quality Assessment

All seven included studies [12,19-24] were retrospective cohort designs with data collection periods spanning 1987-2019, reflecting more than three decades of trauma system and ICU practice evolution. Five studies were conducted in the United States [19-23], one in Japan, and one in Spain. Sample sizes ranged from 26 to 1406 patients. Most were single-center studies [12,21-23]. Three studies [19-21] focused specifically on ICU admission protocols for older adults with rib fractures, whereas four [12,22-24] examined general trauma populations. No study analyzed nonagenarians (≥90 years) as a distinct subgroup. Reported age thresholds varied: four studies included adults aged >65 years, one included those >70 years, one included those >75 years, and one included those >80 years. All seven studies reported mortality data [12,19-24]; five reported intubation rates [19-22,24]; three reported detailed complication data [19-21]; and six reported ICU and hospital length of stay [12,19-23]. Key characteristics of included studies are summarized in Table 1.

**Table 1:** Key data extracted from included studies. Data are presented as numbers (%), mean ± SD, or median (interquartile range, IQR) unless otherwise specified. ISS, injury severity score; LOS, length of stay; ICU, intensive care unit; MVA, motor vehicle accident; M, male; F, female; †, average value; SD, standard deviation

Authors, year, country	Study design, years of data collected, and type of ICU	Sample size, sex	Age included in study, median age	Cause of trauma	Median ISS	Other clinical outcomes	Additional information
Oshima et al. [12], 2018, Japan	Retrospective cohort study, 2013-2016, single center, general ICU	26, M 73.4%, F 24%	>80, 83 (81,85)	Traffic accident (54%), fall (46%)	19 (13,32)	ICU LOS 10 (5,23), hospital LOS 33 (13,57), mortality 11.5%,	Younger cohort mortality 6.2%
Naar et al. [19], 2020, United States	Retrospective cohort study, 2010-2016, multi-center	1445, F 51.6%	>65, >80 = 50.6%, 70-79 = 35.1%, 65-69 = 14.3%	Fall 67.3%, MVA 32.7%	1-9, 50.2%, 10-16, 49.8%	LOS ICU 2 (2,4), LOS hospital 5 (3, 8), intubation 5.7%, complications 6.5%, mortality 3.4%	Major surgical procedure during hospital stay 0.80%
Pyke et al. [20], 2017, United States	Retrospective cohort study 2010-2012 and 2013-2015, single-center trauma ICU	173	>65, pre 77 (71-86) and post 81.5 (74.5-86.5)	Fall 56.6%, cyclist/pedestrian struck 2.3%, MVA 40%	Pre 17 (13-22), post 14 (9-21.5)	LOS ICU pre 6 and post 4, LOS hospital pre 14 and post 10, intubation 17.9%, complications 30%, mortality 5.2%	Surgical procedure during hospital admission 27.7%
Goldstein et al. [21], 2021, United States	Retrospective cohort study, 2016-2019, single center, trauma ICU	101, M 59%	>70, 79 (74,86)	Fall 71%, MVA 20%, pedestrian 7%, assault 2%	9 (8,12)	LOS ICU 5(†), LOS hospital 9(†), intubation 5%, complications 12%, mortality 3%	13% underwent tube thoracostomy
Mulvey et al. [22], 2020, United States	Retrospective cohort study, 2012-2018, single center, trauma ICU	1379, M 52.5%	>65, 78 (71, 85)	Not reported	10 (8-17)	LOS ICU 2 (1-5), LOS hospital 6 (4-11), intubation 4.2%, mortality 10.5%,	Surgical procedure within 24 hours of admission 6.7%
Shabot et al. [23], 1995, United States	Retrospective cohort study, 1987-1990, single center, surgical ICU	99, M 55.6%	>65, 74.1 (†)	Blunt trauma 93.3 %	14.3 (†)	LOS ICU 4.6 (†), LOS hospital 15.3 (†), Mortality 20.2%	Younger cohort mortality 5.8%
Barea-Mendoza et al. [24], 2018, Spain	Retrospective cohort study, 2012-2017, multi-center	1417, M 68.2%, F 31%	>65, 75.5 (70.5, 80.5)	Accidental falls (44.8%)	18 (13-25)	Intubation 61%, mortality 18.2%	Surgical procedure within 24 hours of admission 30.7%, any surgical procedure in hospital stay 12.8%

Risk of bias assessment, conducted independently by two reviewers using ROBINS-I (version 2, 2024) [17] and the SIGN cohort study checklist [18], identified moderate risk of bias in three studies due to limitations in controlling for confounding, incomplete reporting of missing data, or baseline imbalances. One study by Barea-Mendoza et al. [24] that evaluated prognostic model performance (Trauma and ISS vs. Geriatric Trauma Outcome Score) for mortality prediction was judged to be at low risk of bias owing to its focused objective and rigorous statistical design. By contrast, a study by Goldstein et al. [21] evaluating ICU triage criteria for elderly patients with rib fracture was assessed as having serious risk of bias due to absence of multivariate adjustment, reliance on descriptive statistics alone, and unclear outcome definitions. Overall methodological quality of included studies was acceptable; however, the uniformly retrospective designs and limited adjustment for geriatric-specific confounders such as frailty or baseline functional status underscore the need for prospective research in this population. Given the small number of eligible studies and the descriptive nature of our synthesis, all seven studies were retained.

Unplanned ICU Admissions: A Consistent Predictor of Adverse Outcomes

A critical finding across multiple included studies was the consistent association between unplanned ICU admission and substantially worse outcomes. Unplanned ICU admission was defined as either initial management at ward level with subsequent clinical deterioration requiring ICU escalation, or ICU readmission following discharge from ICU to ward level. Naar et al. [19] reported in-hospital mortality of 21.1% for patients with unplanned ICU admission, compared with 3.4% overall mortality in their rib fracture cohort. Mulvey et al. [22] reported mortality of 13.7% for unplanned ICU admission and 10.6% for ICU readmission, compared with 10.5% overall. Pyke et al. [20] similarly found that unplanned admissions were associated with increased intubation rates, respiratory complications, and mortality. This pattern suggests that elderly trauma patients who initially appear stable enough for ward-level care but subsequently deteriorate represent a particularly high-risk subgroup, potentially due to delayed recognition of physiological compromise, inadequate monitoring at ward level, or underlying frailty masking injury severity. These findings have important implications for ICU triage protocols, suggesting that lower thresholds for direct ICU admission or enhanced ward-level monitoring may be warranted for elderly trauma patients.

Mortality

In-hospital mortality was the primary outcome reported across all studies, ranging from 3.0% [21] to 20.2% [23]. A clear pattern emerged when comparing studies by cohort type and injury characteristics. Studies examining rib fracture-specific populations with protocol-driven ICU admission [19-21] reported substantially lower mortality: 3.4% [19], 5.2% [20], and 3% [21]. By contrast, studies examining general trauma populations [12,22-24] reported markedly higher mortality: 10.5% [22], 11.5% [12], 18.2% [24], and 20.2% [23]. This discrepancy likely reflects differences in injury severity, case mix, and patient selection. Rib fracture-specific cohorts had lower median ISSs and represented more homogeneous populations admitted based on specific anatomic criteria (number of rib fractures) rather than hemodynamic instability or multisystem trauma. General trauma cohorts included patients with higher ISS, polytrauma, severe traumatic brain injury (TBI), and heterogeneous admission indications, conferring higher baseline mortality risk.

A sample-size-weighted average in-hospital mortality of 10.5% was calculated across all seven studies. Included studies varied in population age thresholds (≥65 to ≥80 years), injury patterns (isolated rib fractures vs. polytrauma), injury severity (median ISS 9-19), care settings (single-center vs. multicenter; United States, Japan, Spain), and decades of data collection (1987-2019). These sources of heterogeneity preclude interpretation of this weighted average as a precise population estimate; instead, it should be viewed as a descriptive summary indicating the general range of mortality observed across diverse elderly trauma ICU populations. The range of mortality (3.0%-20.2%) and systematic differences between cohort types provide more clinically meaningful information than the summary statistic alone.

All mortality data were reported as in-hospital mortality, with only one study distinguishing between ICU mortality and post-ICU in-hospital mortality [23]. No study reported 30-day, 90-day, or 1-year mortality. As described above, unplanned ICU admission was consistently associated with elevated mortality across studies. Key data extracted from included studies are displayed in Table 1.

Injury Severity

**Figure 2:**
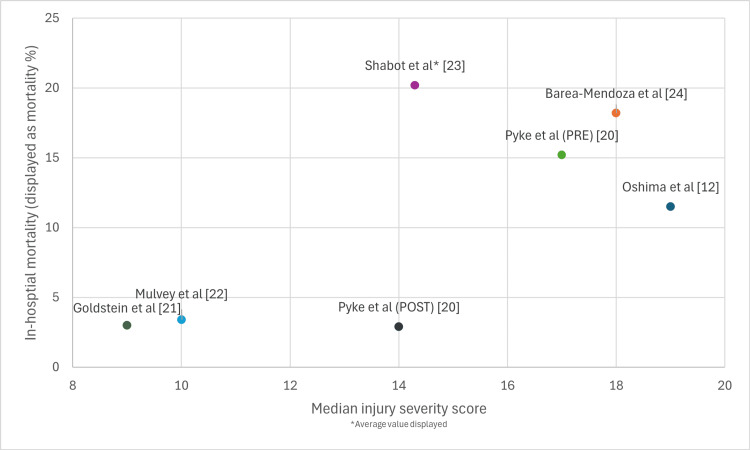
Mortality and injury severity score.

Median ISS varied substantially and demonstrated clear association with cohort type. Studies examining rib fracture-specific populations reported median ISS of 9 (IQR 8-12) [21] or ISS ranges predominantly between 1 and 16, reflecting isolated thoracic trauma or limited additional injuries. Pyke et al. [20], while focusing on blunt thoracic trauma, reported higher median ISS (14-17 across two time periods), as their cohort encompassed broader thoracic injuries beyond isolated rib fractures. Studies examining general trauma populations reported notably higher ISS: median 10 (IQR 8-17), mean 14.3, median 18 (IQR 13-25), and median 19 (IQR 13-32). Higher ISS was consistently associated with increased complications, prolonged ICU stay, and elevated mortality across studies, underscoring that injury severity rather than age alone is a critical determinant of outcomes in elderly trauma patients admitted to ICU. The relationship between mortality and injury severity across included studies is presented in Figure 2.

Complications and Intubation

Intubation rates varied dramatically and demonstrated clear association with cohort type and injury severity. Rib fracture-specific cohorts reported low intubation rates: 5.0%, 5.7%, and 17.9%. General trauma cohorts reported substantially higher rates: 4.2% (noting this study's population had lower median ISS) and 61.0%. The exceptionally high intubation rate reported by Barea-Mendoza et al. [24] reflected their cohort composition: 71% had TBI as the primary injury, with median ISS 18 (IQR 13-25), representing the most severely injured population among included studies. This pattern illustrates that injury pattern, particularly TBI versus isolated thoracic injury, is a more important determinant of intubation requirement than age itself within the elderly range studied (≥65-80 years).

Respiratory complications, including pneumonia, respiratory failure, and pleural effusions, were the most frequently reported across studies [19-21]. However, complication reporting was inconsistent, with only three studies [19-21] providing detailed complication data. Among general trauma cohorts, Mulvey et al. [22] reported sepsis in 9.5%, acute kidney injury in 5.3%, and cardiac dysrhythmia in 26.3%, with higher rates observed among patients experiencing unplanned ICU admission. Patients requiring mechanical ventilation had prolonged ICU stays and increased mortality [22].

Trauma Mechanisms

Falls were overwhelmingly the predominant trauma mechanism across all included studies, accounting for 44.8% [24] to 67.3% [19] of cases, with Goldstein et al. [21] reporting the highest proportion at 71%. Motor vehicle collisions constituted the second most common mechanism, ranging from 20% [21] to 54% [12], with the highest rate observed in the Japanese cohort, potentially reflecting regional differences in trauma epidemiology or road safety patterns. Pedestrian injuries and assaults accounted for smaller proportions of admissions.

ICU Length of Stay

ICU length of stay demonstrated substantial variation associated with cohort type, injury severity, and complications. Rib fracture-specific cohorts with protocol-driven ICU admission reported the shortest stays: median 2 days (IQR 2-4) [19] and mean 4-6 days. These populations were admitted primarily for pain management, pulmonary physiotherapy, and respiratory monitoring rather than for hemodynamic instability or mechanical ventilation. Notably, Goldstein et al. [21] reported an over-triage rate of 87%, indicating that many patients admitted to ICU based on anatomic injury criteria (number of rib fractures) did not ultimately require ICU-level interventions.

General trauma cohorts reported longer ICU stays: median 2 days (IQR 1-5), mean 4.6 days, and median 10 days (IQR 5-23). The longest stays were observed in patients requiring mechanical ventilation due to severe injuries and high ISS [12,23]. Oshima et al. [12], examining the oldest cohort (≥80 years) with the highest median ISS of 19, reported the longest median ICU stay (10 days), illustrating the combined impact of advanced age within the elderly spectrum and high injury severity. Unplanned ICU admissions and complications significantly prolonged ICU stay, leading to increased healthcare resource utilization [19,20,22]. One study [21] found an over-triage rate of 87% into the ICU for older adult trauma patients with simple rib fracture.

Surgical Interventions

Five studies reported surgical intervention rates [19-22,24], which varied markedly: 0.8%, 6.7% within 24 hours, 27.7%, and 30.7% within 24 hours. The lowest rates were observed in rib fracture cohorts, where conservative management with analgesia and respiratory support predominates. The highest rate (30.7% within 24 hours) was reported by Barea-Mendoza et al. [24] and reflected their cohort with 71% TBI requiring neurosurgical interventions (craniotomy, ventriculostomy, intracranial pressure monitoring). Chest drainage and thoracentesis were the most frequently reported procedures in rib fracture cohorts [19-21]. Other procedures included major orthopedic surgery, rib fixation, tracheostomy, and neurosurgical interventions. A detailed summary of surgical procedures is presented in Table 2.

**Table 2:** Summary of reported surgical procedures across studies, expressed as a percentage of the total study cohort.

Study	Procedure	Reported value
Pyke et al. [20]	Major surgery	27.4%
	Major orthopedic surgery	19.4%
	Craniotomy/ventriculostomy/intracerebral pressure monitor	3.9%
	Major thoracic surgery	0.6%
	Major abdominal surgery	2.9%
	Tracheostomy	7.6%
Naar et al. [19]	Thoracentesis/chest tube	4.6%
	Video-assisted thoracic surgery	0.2%
	Decortication	0.1%
	Chest operation	0.2%
	Rib fixation	0.4%
	Epidural placement	5.0%
Barea-Mendoza et al. [24]	Surgical procedure within 24 hours	30.7%
	Any surgical procedure during hospitalization	12.8%
Goldstein et al. [21]	Thoracentesis/chest tube	13%
Mulvey et al. [22]	Surgical procedure within 24 hours	6.7%

Synthesis Across Studies

Synthesizing findings across included studies, a consistent pattern emerges: outcomes in elderly trauma ICU patients are primarily determined by injury severity, injury pattern (particularly TBI vs. isolated thoracic injury), and ICU admission pathway (planned vs. unplanned) rather than by age alone within the broad elderly category studied (≥65 to 80 years). Rib fracture-specific cohorts with protocol-driven ICU admission demonstrate low mortality (3%-5%), low intubation rates (5%-18%), and short ICU stays (2-6 days), but high rates of potential over-triage, suggesting that many patients might be managed safely at lower levels of care with appropriate monitoring. General trauma cohorts with higher ISS, polytrauma, and particularly TBI demonstrate substantially higher mortality (10%-20%), intubation rates (up to 61%), and ICU length of stay (up to 10 days). Across all cohort types, unplanned ICU admission consistently predicts worse outcomes than protocol-driven or initially planned ICU admission, highlighting the importance of accurate early triage and the risks associated with delayed recognition of physiological deterioration in elderly trauma patients.

Discussion

This scoping review reveals a fundamental gap in the trauma and critical care literature: no published studies have examined nonagenarians (≥90 years) admitted to ICU following traumatic injury as a distinct cohort. Although seven retrospective cohort studies reported outcomes for elderly trauma patients, all aggregated populations across broad age ranges (≥65 to ≥80 years) without age-stratified subgroup analyses that would isolate the oldest adults. Only one study specifically examined patients aged over 80 years, comparing them with younger trauma cohorts but not extending analysis to nonagenarians or reporting long-term functional outcomes. This absence of nonagenarian-specific evidence represents a critical knowledge deficit given the rapid demographic expansion of this population and their increasing use of ICU-level care.

Interpretation of Findings in the Context of Study Limitations

The interpretation of our findings must be carefully contextualized within the substantial limitations of the included primary studies. All seven were retrospective cohort designs relying on administrative databases or trauma registries that lack detailed information on pre-injury functional status, frailty, baseline cognition, and pre-existing functional dependencies, all critical factors that influence both survival and the quality of survival in elderly trauma patients. The absence of such data means we cannot determine whether survivors returned to baseline function, experienced significant new disability, or required permanent institutional care. A 75-year-old patient who survives ICU admission with 10.5% descriptive average mortality but subsequently requires long-term mechanical ventilation, permanent nursing home placement, and complete loss of independence represents a vastly different outcome from a 75-year-old who returns home with full functional recovery, yet these profoundly different trajectories are indistinguishable in the current evidence base.

Furthermore, the heterogeneity in age thresholds (≥65 to ≥80 years) across studies precludes age-specific interpretation. Outcomes reported for populations with median ages in the early-to-mid 70s cannot be assumed to apply to nonagenarians, given the well documented exponential increases in frailty prevalence, comorbidity burden, and physiological vulnerability with each decade of advancing age. The mortality rates, complication rates, and ICU length of stay reported in this review likely substantially underestimate the true burden for the oldest patients (≥90 years) if they were analyzed separately. Conversely, the broad age inclusion may mask important heterogeneity within the elderly population, wherein relatively young elderly patients (65-75 years) with preserved function may have substantially better outcomes than the oldest elderly with frailty.

The retrospective designs also introduce selection bias that cannot be quantified. Clinicians making ICU admission decisions for elderly trauma patients engage in complex, multifactorial decision-making that incorporates not only injury severity but also perceived baseline function, family preferences, advance care planning documentation, and subjective assessments of likelihood of meaningful recovery. Patients admitted to ICU therefore represent a selected subgroup deemed appropriate candidates for life-sustaining intervention, whereas potentially similarly injured patients managed palliatively or with comfort measures only are not captured in these cohorts. This selection bias means that reported ICU outcomes cannot inform the broader question of "what happens to elderly patients with severe trauma" but only "what happens to elderly patients with severe trauma who are selected for ICU admission", a critically important distinction for resource allocation, prognostication, and ethical decision-making. The outcomes we observe may therefore represent a best-case scenario applicable only to selected patients rather than generalizable estimates for all elderly trauma patients with ICU-level illness.

Temporal Heterogeneity and Changes in Trauma Systems

The temporal heterogeneity of included studies warrants explicit consideration. Data collection spanned from 1987-1990 to 2012-2019, encompassing more than three decades during which trauma care and ICU practice evolved substantially. Relevant advances include: implementation of inclusive trauma systems with regionalization, interfacility transfer protocols, and trauma center verification; development of geriatric-specific trauma protocols and dedicated geriatric trauma services in some centers; advances in CT imaging enabling rapid, detailed injury characterization; evolution of mechanical ventilation strategies including lung-protective ventilation and increased use of non-invasive ventilation; modern sepsis management with early recognition, protocolized resuscitation, and antimicrobial stewardship; multimodal analgesia and regional anesthesia techniques for rib fracture management; early mobilization protocols and systematic ICU delirium prevention strategies; and formal integration of palliative care and goals-of-care discussions for critically ill elderly patients.

Shabot et al. [23], reporting data from 1987-1990, documented the highest mortality (20.2%) among included studies. While this finding reflects their patient population characteristics and injury severity distribution, it may also partly reflect ICU care capabilities of that era compared with contemporary practice. Conversely, more recent studies reporting lower mortality rates may benefit from modern trauma systems organization and ICU interventions unavailable to earlier cohorts. This temporal confounding cannot be quantified or adjusted for retrospectively but must be considered when interpreting aggregate findings. The sample-size-weighted average mortality of 10.5% calculated across studies spanning three decades likely obscures important temporal trends and may not accurately represent outcomes achievable with current evidence-based practices.

Functional Outcomes: The Most Critical Evidence Gap

Only four studies [19-21,23] reported limited data on discharge destination (home vs. rehabilitation facility vs. long-term care), and none reported functional status, ability to perform activities of daily living, cognitive outcomes, quality of life, or caregiver burden at discharge or at any time point during follow-up. This represents arguably the most significant gap in the current evidence base. For elderly patients and their families facing decisions about ICU admission after trauma, survival alone is rarely the outcome of primary importance. Rather, survival with meaningful functional recovery, preservation of independence, maintenance of cognitive function, and acceptable quality of life are the patient-centered outcomes that inform shared decision-making and determine whether aggressive ICU intervention aligns with patients' values and goals.

The absence of such data means that clinicians cannot provide evidence-based counselling regarding realistic expectations for post-ICU functional trajectories, cannot stratify patients by likelihood of returning to baseline independence, and cannot adequately inform family discussions regarding the risks and benefits of ICU admission relative to comfort-focused care. This limitation is compounded by the uniformly short outcome assessment windows: all studies reported in-hospital mortality or at most discharge destination, with no information on 30-day, 90-day, or 1-year mortality; no data on hospital readmission rates; no assessment of whether patients surviving to discharge subsequently experience accelerated functional decline or death; and no evaluation of long-term survival, disability-free survival, or health-related quality of life. For populations with median ages in the 70s to early 80s, understanding outcomes extending beyond the index hospitalization is essential for determining whether ICU intervention after trauma truly extends life with acceptable quality or merely postpones mortality with an intervening period of disability, dependence, and suffering.

Injury Pattern Specificity and Knowledge Gaps

Three included studies [19-21] focused exclusively on rib fracture populations, reflecting the historical research emphasis on blunt thoracic trauma in elderly patients and the resultant development of rib fracture-based ICU admission protocols. By contrast, only one study included a substantial proportion of patients with TBI (71% of their cohort), who demonstrated markedly different outcomes including exceptionally high intubation rates (61%) and elevated mortality (18.2%). This striking difference underscores that injury pattern, and specifically the presence of significant TBI, is a more important determinant of ICU resource needs and mortality risk than age alone within the elderly spectrum studied (≥65 to 80 years). However, the near-absence of literature on elderly ICU trauma patients with other injury patterns (severe abdominal trauma, pelvic fractures, major vascular injuries, polytrauma) limits our ability to develop evidence-based, injury-specific ICU triage criteria for elderly patients beyond rib fractures. Similarly, although four studies documented comorbidity prevalence, none analyzed the relationship between specific comorbidities (or comorbidity burden) and mortality, and no study systematically measured or reported frailty despite its established prognostic importance in elderly critical illness.

Unplanned ICU Admission: Implications for Practice

The consistent finding across multiple studies that unplanned ICU admission, whether delayed escalation from ward-level care or readmission after ICU discharge, predicts substantially worse outcomes (mortality 13.7-21.1%) compared with planned direct ICU admission has important implications. It suggests either that elderly trauma patients who initially appear hemodynamically stable may harbor physiological fragility that becomes clinically apparent only after a delay, or that current ward-level monitoring is inadequate to detect early decompensation in this population. These findings support consideration of lower thresholds for direct ICU admission or implementation of enhanced monitoring protocols (e.g., intermediate care units, continuous vital sign monitoring, geriatric trauma consultation) for elderly trauma patients triaged to ward-level care.

Priorities for Future Research

Future research should address the identified evidence gaps through a prioritized approach. The highest priority is prospective cohort studies specifically examining trauma patients aged ≥90 years admitted to ICU, with direct comparison to octogenarian (80-89 years) and younger elderly (70-79 years) cohorts using age-stratified analyses. Such studies must employ standardized, patient-centered outcome measures including: mortality at multiple time points (in-hospital, 30-day, 90-day, 6-month, 1-year); functional status at discharge and during follow-up using validated instruments (Barthel Index, Katz Activities of Daily Living scale); return to pre-injury level of independence; health-related quality of life (EQ-5D or SF-36); discharge destination and requirement for new post-acute care services; and hospital readmission rates. Systematic measurement and reporting of frailty using validated instruments (Clinical Frailty Scale, Fried phenotype, or deficit-accumulation indices) as an independent predictor of outcomes, adjusted for injury severity and comorbidities, is essential and will help determine whether frailty adds prognostic information beyond chronological age and ISS.

Research examining injury patterns beyond rib fractures, particularly TBI, abdominal trauma, pelvic trauma, and polytrauma, in the oldest adults is urgently needed to develop evidence-based, injury-specific ICU triage and management protocols. Studies should also prospectively identify risk factors for unplanned ICU admission among elderly trauma patients initially managed at ward level, develop and validate early warning systems or enhanced monitoring protocols, and evaluate whether lower thresholds for ICU admission improve outcomes or represent over-treatment. Finally, the trauma and critical care research community should establish consensus core outcome sets for elderly trauma research, ensuring that all future studies report standardized mortality time points, functional outcomes using validated instruments, quality of life, discharge disposition, and age-stratified subgroup analyses in at least five-year or 10-year bands extending to ≥90 years. Such standardization will enable future systematic reviews with meta-analysis and meaningful cross-study comparisons that are currently impossible.

Limitations of Primary Studies

The included studies have several important limitations affecting interpretation. All were retrospective cohort designs relying on trauma registries or administrative databases, introducing risks of selection bias, incomplete data capture, unmeasured confounding, and inability to assess outcomes not systematically recorded. These studies lack information on pre-injury frailty, functional status, cognition, advance care planning, and ICU admission decision-making processes, factors that critically influence both who receives ICU care and how outcomes should be interpreted. No study analyzed nonagenarians as a distinct cohort, limiting direct applicability to this target population. Included studies varied substantially in age thresholds, injury types, injury severity, care settings, and decades of data collection (1987-2019), introducing heterogeneity that limits generalizability. Outcome reporting was restricted to short-term metrics (in-hospital mortality, ICU length of stay), with long-term survival, functional outcomes, quality of life, and post-discharge trajectories rarely reported. Finally, studies spanning multiple decades reflect different eras of trauma system organization and ICU practice, with temporal changes in care potentially influencing outcomes in ways that cannot be adjusted for retrospectively.

Limitations of the Scoping Review Methodology

Beyond limitations of primary studies, this scoping review has methodological limitations. No protocol was prospectively registered, introducing potential for selection or reporting bias despite adherence to established scoping review frameworks and dual independent screening. The database search was limited to MEDLINE and the Cochrane Library; additional databases (Embase, Scopus, Web of Science, CINAHL) might have identified further studies, particularly international or specialty journal publications. Grey literature searching via general search engines has inherent reproducibility limitations. Data extraction by a single reviewer with verification by a second introduces greater error risk than fully independent dual extraction. The analytical approach was purely descriptive without quantitative synthesis or formal heterogeneity assessment; while appropriate for scoping review objectives, this precludes pooled effect estimates. The sample-size-weighted average mortality should be interpreted as a descriptive summary across heterogeneous populations rather than a precise meta-analytic estimate. Finally, deliberately broad inclusion criteria (age ≥65 years, any trauma type) maximized evidence capture but resulted in substantial heterogeneity limiting clinical specificity for the target nonagenarian population.

## Conclusions

This scoping review reveals that current evidence on ICU outcomes following trauma in elderly patients derives exclusively from heterogeneous retrospective cohorts aged ≥65 to 80+ years and provides no nonagenarian-specific data. Within these broader elderly ICU trauma populations, several consistent patterns emerged: in-hospital mortality ranged from 3.9% to 20.2% (descriptive weighted average 10.5%) with substantial variation by injury type and severity; higher ISS and unplanned ICU admission were consistently associated with increased mortality; falls were the predominant trauma mechanism (45%-67%); and respiratory complications were common, particularly among patients with high ISS or TBI. However, the absence of dedicated analyses for patients aged ≥90 years, the near-total lack of long-term functional and quality-of-life outcomes, and the retrospective design of all available studies substantially limit inferences for nonagenarians and preclude evidence-based prognostication for the oldest trauma patients considering ICU admission.

Based on available evidence from younger elderly cohorts (≥65 to 80 years), recognizing that extrapolation to nonagenarians involves uncertainty, several practice considerations emerge. Early, protocol-driven ICU triage for elderly trauma patients with high ISS or high-risk injury patterns may be preferable to initial ward-level management given the consistent association between unplanned ICU admission and adverse outcomes. Development of institutional ICU admission guidelines for elderly trauma should incorporate age-specific considerations and injury pattern-specific criteria, recognizing that rib fracture protocols have some evidence base whereas protocols for TBI, abdominal trauma, and polytrauma remain underdeveloped. Enhanced ward-level monitoring or lower thresholds for ICU admission warrant consideration given the poor outcomes associated with delayed recognition of deterioration.

Future research must prioritize prospective, age-stratified cohort studies including nonagenarians with comprehensive assessment of functional outcomes, frailty, and quality of life using standardized instruments; examination of injury patterns beyond rib fractures; and reporting of outcomes extending beyond hospital discharge to capture longer-term survival and functional trajectories. Only through such research can the trauma and critical care community provide clinicians, patients, and families with the evidence necessary for informed, shared decision-making regarding ICU admission and goals of care for the oldest adults following traumatic injury. Given the rapid demographic expansion of the nonagenarian population and intensifying constraints on critical care resources, generating this evidence is both a scientific priority and an ethical imperative.

## Appendices

Appendix 

**Table 3:** Search strategy Related terms (e.g. “trauma” OR “wounds and injuries”) were grouped conceptually within the search strategy to maximize sensitivity; search lines are reported verbatim to allow reproducibility.

Databases searched: MEDLINE (via Ovid), Cochrane Library. Initial search: February 10, 2025. Subsequent search: April 10, 2025.	
1	Nonagenarian.mp. or exp Nonagenarians/
2	Elderly.mp. or Aged/
3	(Aged, 80 or over).mp. [mp=title, book title, abstract, original title, name of substance word, subject heading word, floating sub-heading word, keyword heading word, organism supplementary concept word, protocol supplementary concept word, rare disease supplementary concept word, unique identifier, synonyms, population supplementary concept word, anatomy supplementary concept word]
4	(Aged, 90 or over).mp. [mp=title, book title, abstract, original title, name of substance word, subject heading word, floating sub-heading word, keyword heading word, organism supplementary concept word, protocol supplementary concept word, rare disease supplementary concept word, unique identifier, synonyms, population supplementary concept word, anatomy supplementary concept word]
5	Geriatric.mp. or exp Geriatrics/ or exp Aged/
6	Intensive Care Units/ or ICU admission.mp. or Critical Illness/
7	Critical care.mp. or Critical Care/
8	"Wounds and Injuries"/ or traumatic injury.mp.
9	Trauma.mp. or "Wounds and Injuries"/
10	Patient admission.mp. or Patient Admission/
11	1 or 2 or 3 or 4 or 5
12	8 or 9
13	6 or 7
14	11 and 12
15	13 and 14
16	10 and 15
